# Supplementary material for: COZOID: contact zone identifier for visual analysis of protein-protein interactions
Source: BMC Bioinformatics. 2018 Apr 6;19:125. doi: 10.1186/s12859-018-2113-6 (PMC5889581; doi:10.1186/s12859-018-2113-6)
Supplement: Supplementary file 2 — Software build. Executable binary file of the software tool. (ZIP 118,784 kb) [file 12859_2018_2113_MOESM2_ESM.zip › cozoid/java/docs/beansbinding-1.2.1-doc.zip!/org/jdesktop/el/impl/lang/package-frame.html]

org.jdesktop.el.impl.lang (Beans Binding)


org.jdesktop.el.impl.lang

Classes 
  
ELArithmetic
  
ELArithmetic.BigDecimalDelegate
  
ELArithmetic.BigIntegerDelegate
  
ELArithmetic.DoubleDelegate
  
ELArithmetic.LongDelegate
  
ELSupport
  
EvaluationContext
  
ExpressionBuilder
  
FunctionMapperFactory
  
FunctionMapperImpl
  
FunctionMapperImpl.Function
  
VariableMapperFactory
  
VariableMapperImpl
